# A Member of the Hospital Nursing Staff Articulates

**Published:** 1918-09-28

**Authors:** 


### A Member of the Hospital Nursing Staff Articulates.

*To the Editor of* The Hospital.

Sir,—I have had the honour of being on the London Hospital nursing staff for the past sixteen years and would like to answer Dr. Chapple's unjust criticism of the management and treatment of the nursing staff of this hospital.

I entered the London Hospital as a probationer, having had no previous experience in nursing. At the end of two years I gained my certificate, and from then until the present day I have received, and so too has the hospital, many reports and letters of appreciation and thanks from patients, surgeons, and physicians for the services of the "trained nurse" who owed all her skill to the training she received at the London Hospital. There are hundreds of "Londoners" who could and would say the same.

Our salary in these hard cash days may seem inadequate to a business man, but a woman needs more than mere £ s. d., and she gets more at the London Hospital.

Each nurse has her own separate room. She gets her full regular off-duty time and her day off. On her "days off" she can have her breakfast in bed, and if she does not wish to go out she can attend meals in the dining-room or have them in her own room. When she is sick, she is nursed and cared for, even during her convalescence too. If she is poor, or has no restful home to go to, the hospital arranges a convalescent home for her. She has uniform provided and her laundry done free.

She has her salary paid during her sick leave and also during special leave, unless it is essential for her to be away for a long period, when indefinite leave is granted.

At the end of eighteen years' service she gets a pension, which is full pay for life.

I can tell Dr. Chapple why the nurses stay so long at the "London," and why it was possible to send over one hundred private staff nurses to nurse our brave men in France, Egypt, and Palestine, and why many of them are looking forward to the day when they can return to their old hospital.

It is because the hospital is not more interested in its banking account than in its nurses' comfort, and consequently we are proud to do all we can to help the hospital in return for all it does for us.

A Member of the London Hospital Nursing Staff.

